# Antibacterial Cleaning Products and Drug Resistance

**DOI:** 10.3201/eid1110.041276

**Published:** 2005-10

**Authors:** Allison E. Aiello, Bonnie Marshall, Stuart B. Levy, Phyllis Della-Latta, Susan X. Lin, Elaine Larson

**Affiliations:** * University of Michigan School of Public Health, Ann Arbor, Michigan, USA; † Tufts University School of Medicine, Boston, Massachusetts, USA; ‡ Columbia University, New York, New York, USA

**Keywords:** Antibacterial products, triclosan, antibiotic resistance, antimicrobial drug resistance, household, research

## Abstract

We examined whether household use of antibacterial cleaning and hygiene products is an emerging risk factor for carriage of antimicrobial drug–resistant bacteria on hands of household members. Households (N = 224) were randomized to use of antibacterial or nonantibacterial cleaning and hygiene products for 1 year. Logistic regression was used to assess the influence of antibacterial product use in homes. Antibacterial product use did not lead to a significant increase in antimicrobial drug resistance after 1 year (odds ratio 1.33, 95% confidence interval 0.74–2.41), nor did it have an effect on bacterial susceptibility to triclosan. However, more extensive and longer term use of triclosan might provide a suitable environment for emergence of resistant species. Further research on this issue is needed.

## Abstract 2

Levels of antimicrobial drug resistance did not differ significantly between persons in households that used antibacterial cleaning and hygiene products and those that did not.

Concern is growing over the use of household cleaning and hygiene products labeled as antibacterial as a result of laboratory data showing a link between exposure to ingredients in these products, particularly triclosan, and emergence of antimicrobial drug resistance (*1**–**3*). This study aimed to determine whether home use of antibacterial cleaning and hygiene products (including use of a handwashing soap containing 0.2% triclosan) or other potential risk factors was associated with carriage of antimicrobial drug–resistant bacteria on household members’ hands. We also assessed the association of these antibacterial products with carriage of organisms with reduced susceptibility to triclosan.

## Materials and Methods

### Study Population

**Figure 1:**
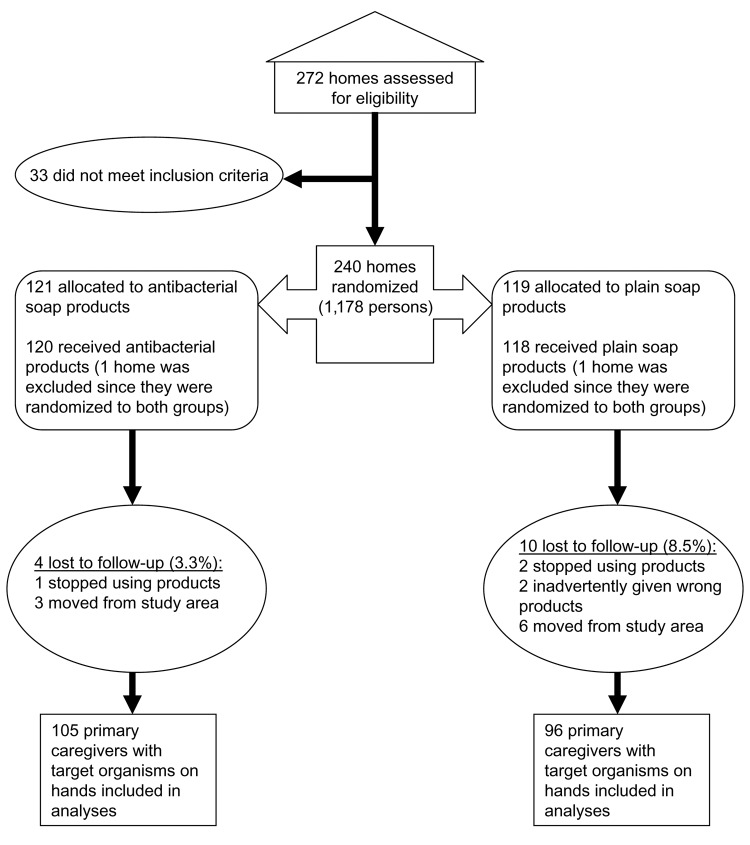
Flow chart for randomized trial. After randomization and loss to follow-up, the remaining study participants who carried target organisms were included in the logistic regression analyses.

The data for this study were collected as part of a double-masked and randomized home intervention trial (*4*); participant enrollment began in October 2000, and follow-up occurred for a 12-month period. The methods and randomization procedures for this study have been reported elsewhere (*5*). A total of 238 households were recruited at baseline; 224 households completed the entire 1-year follow-up (Figure 1). The study was approved by Columbia University Medical Center Institutional Review Board.

### Intervention Methods

Households were supplied with over-the counter, generically repackaged consumer cleaning and personal hygiene products free of charge on a monthly or as-needed basis. Households randomly assigned to use antibacterial products received the following: 1) liquid handwashing soap containing 0.2% triclosan, 2) liquid kitchen spray and liquid all-purpose cleaner for hard surfaces that contained a quaternary ammonium component, and 3) oxygenated bleach laundry detergent. Households randomly assigned to the nonantibacterial group received the same products but without antibacterial ingredients. Both groups received the same nonantibacterial liquid dishwashing detergent and bars of body soap to control for potential use of other products that might contain antibacterial ingredients. Study participants were required to use only assigned home hygiene products and were asked not to change any of their normal hygiene practices. Participants, interviewers, and study coordinators were blinded to brand names and ingredients in all products. Adherence to product treatment group was assessed monthly, and products were weighed during each visit to monitor compliance. Households were immediately dropped from the study if they did not adhere to randomized treatments.

### Data Collection

At baseline, and quarterly during the 1-year period, a trained interviewer collected demographic information from the person self-identified as the primary caregiver in the household. The baseline interview determined the type of handwashing soap, hygiene, and cleaning products that were used before randomization into the study (i.e., the brand and whether or not the ingredients were labeled as antibacterial). The baseline and quarterly assessment forms provided information such as the number and age of household members, childcare attendance, symptoms of infectious illnesses (fever, diarrhea, sore throat, vomiting, conjunctivitis, skin boils, runny nose), antimicrobial drug use, chronic diseases, self-rated health, birthplace, travel outside of the United States, and occupation. In addition, reported number of handwashes per day by the primary caregiver and a timed observation of the handwash before hand culturing were gathered.

The hands of the primary caregiver were cultured during the home visit at baseline and at the end of the 12-month period before and after washing with the assigned liquid handwashing product. The trained data collector used a coin flip to choose the test hand, which was then inserted into a sterile polyethylene bag containing 50 mL culture medium (0.075 mol/L phosphate buffer, pH 7.9, containing 0.1% polysorbate 80). The hand was massaged for 1 min through the wall of the bag containing culture medium. Only postwash samples were used in analyses since they were considered to be representative of normal versus transient flora found on hands.

### Laboratory Methods

The laboratory methods for this study have been described previously (*5**,**6*). The microbiologic analysis and antimicrobial drug–susceptibility testing were conducted at New York Presbyterian Hospital, Columbia University Medical Center, New York. Selective media were used to isolate gram-positive cocci, gram-negative bacteria (GNB), *Staphylococcus aureus*, and enterococci.

Only clinically important bacterial species that were prevalent (species with >38 isolates recovered at baseline and end of year combined) on the hands of homemakers were selected for susceptibility analyses (*7**,**8*). These included the following GNB: *Acinetobacter baumannii*, *A. lwoffi*, *Enterobacter agglomerans*, *E. cloacae*, *Klebsiella pneumoniae*, and *Pseudomonas fluorescens*/*putida*; and the following gram-positive staphylococci: *S. aureus*, *S. warneri*, *S. epidermidis*, and *S. capitis*. Therefore, only persons who were carrying at least 1 of these organisms on their hands were included in the final analyses (N = 164 at baseline and N = 201 at year-end). No significant differences were noted between the measured demographic characteristics (Tables 1 and 2 for listing of demographics) among those included in the final analyses versus those excluded (all p>0.10).

**Table 1:** Proportion of all study participants with baseline characteristics

Characteristics*	Nonantibacterial groups† (N = 118), %	Antibacterial groups† (N = 120), %
Primary caregiver		
Male primary caregivers	4.2	4.2
Caregivers born outside of United States	94.1	98.3
Caregivers with high CFU counts on hands‡	35.8	39.4
Household		
Antibacterial cleaning and hygiene products used prebaseline	41.5	40.0
Characteristics reported for >1 members of the household		
Child in daycare	15.9	17.8
Chronic illness	39.0	37.0
Chronic illness or fair to poor health	61.0	55.8
Symptoms of infection in past 30 days	54.2	54.2
Use of antimicrobial agents in past 30 days§	11.9	11.7
Traveled outside United States in past month	12.8	12.5
Healthcare or daycare occupation	41.0	45.0

*No significant differences in demographic characteristics between persons with or without available cultures or between participants with or without gram-negative bacteria or staphylococci of interest were noted in this study (all p>0.10). †No significant differences between the antibacterial and nonantibacterial users in any of the characteristics measured were noted (all p>0.05). ‡Culture information was not available at baseline for 20 study participants. High counts were determined by whether the participant had a CFU above the mean for the entire group. §Information on use of antimicrobial agents use was only gathered from study participants reporting infectious symptoms. Therefore, all persons reporting no infectious symptoms were coded as having "no reported antibiotic use."

**Table 2:** Mean values for baseline or year-end characteristics of study participants

Characteristic	Nonantibacterial group* (N = 118)		Antibacterial group* (N = 120)	
	Mean	SD	Mean	SD
Primary caregiver				
Age (y) of primary caregiver (baseline)	34.6	10.0	33	8.1
No. of daily washes (reported)				
Baseline	13.3	9.8	11.6	7.1
End of year	11.6	6.3	10.3	5.1
Length(s) of handwash (observed)				
Baseline	15.5	9.4	16.4	9.7
End of year	18.7	8.3	18.5	8.3
Household				
Age (y) of all household members combined (baseline)	20.1	4.9	20.0	5.9
No. of children <5 y in home (baseline)	1.5	0.6	1.5	0.7
No. of persons in household (baseline)	5.0	1.5	5.0	1.8

*No significant differences were observed between the antibacterial and nonantibacterial product users in any of the characteristics measured (all p>0.05).

Bacterial isolates were tested against a panel of antimicrobial agents by using MicroScan WalkAway 96 SI (Dade Behring, Deerfield, IL, USA). Using the recommendations of the Clinical and Laboratory Standards Institute (formerly NCCLS), we classified antimicrobial drug susceptibility as resistant, intermediate, or susceptible to a particular antimicrobial agent (*9*). Organisms that tested as either resistant or intermediately resistant to antimicrobial agents were classified as "antibiotic resistant" (*10*). The selection of antimicrobial agents to be tested for each organism was based on clinical applicability of the antimicrobial drug and consistency with earlier studies that examined a link between triclosan and antimicrobial drug resistance (*11**–**14*). GNB were tested against several antimicrobial agents, and staphylococci were tested against oxacillin to indicate methicillin resistance. For analytic purposes, GNB species were classified as resistant if a given isolate was resistant to >1 antimicrobial agent(s).

Triclosan susceptibility was examined at Tufts University School of Medicine, Boston, Massachusetts, by using a modified NCCLS agar dilution method (*10*). Minimum inhibitory concentration (MIC) was defined as the lowest dilution of triclosan that inhibited visible growth. A detailed description of antimicrobial drug and triclosan testing, including controls used and MIC distribution for each organism, has been described previously (*6*). Since data from the literature regarding triclosan susceptibility testing are sparse and provide no standardized breakpoints (*6*), we dichotomized triclosan MIC values for each isolate by using the median MIC as a cutoff; low MIC represents less than or equal to the median value and high MIC indicates greater than the median value.

### Analytic Methods

First, chi-square and Student *t* tests were used to compare demographic characteristics of antibacterial and nonantibacterial users. Next, chi-square tests were used to compare the overall proportion of antimicrobial drug–resistant isolates found on the hands of the antibacterial and nonantibacterial groups. Finally, multivariate logistic regression analyses were conducted to examine the relationship between antibacterial product use and 2 separate outcome variables: antimicrobial drug resistance (measured by the presence of >1 antimicrobial drug–resistant species on the hand) and increased triclosan MICs (measured by the presence of >1 species exhibiting a triclosan MIC above the median value).

Each potential covariate (i.e., characteristics of the household and primary caregiver) and our 2 outcome variables were examined in univariate analyses to establish criteria for inclusion in final multivariate models by using a p value <0.05 as the cutoff. Covariates meeting the cutoff criteria were included in multivariate models along with the main effect of the randomized treatment (i.e., antibacterial versus nonantibacterial product use). Analyses were conducted separately for baseline and after 1 year of study participation. Unadjusted and adjusted odds ratios (OR) and 95% confidence intervals (CIs) were generated from logistic regression analyses by using SPSS V.10 (SPSS Inc., Chicago, IL, USA).

## Results

**Figure 2:**
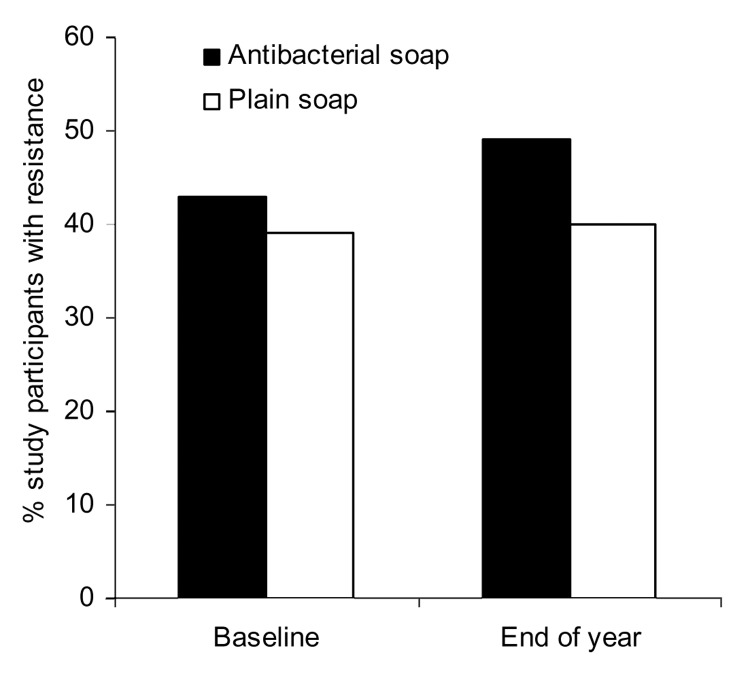
Proportion of study participants with >1 bacterial species resistant to an antimicrobial agent on their hands. In the group that used antibacterial products, 82 and 105 hand samples were available at baseline and at year-end, respectively. In the group that used nonantibacterial products (i.e., plain soap), 82 and 96 hand samples were available at baseline and at year-end, respectively.

**Figure A1:**
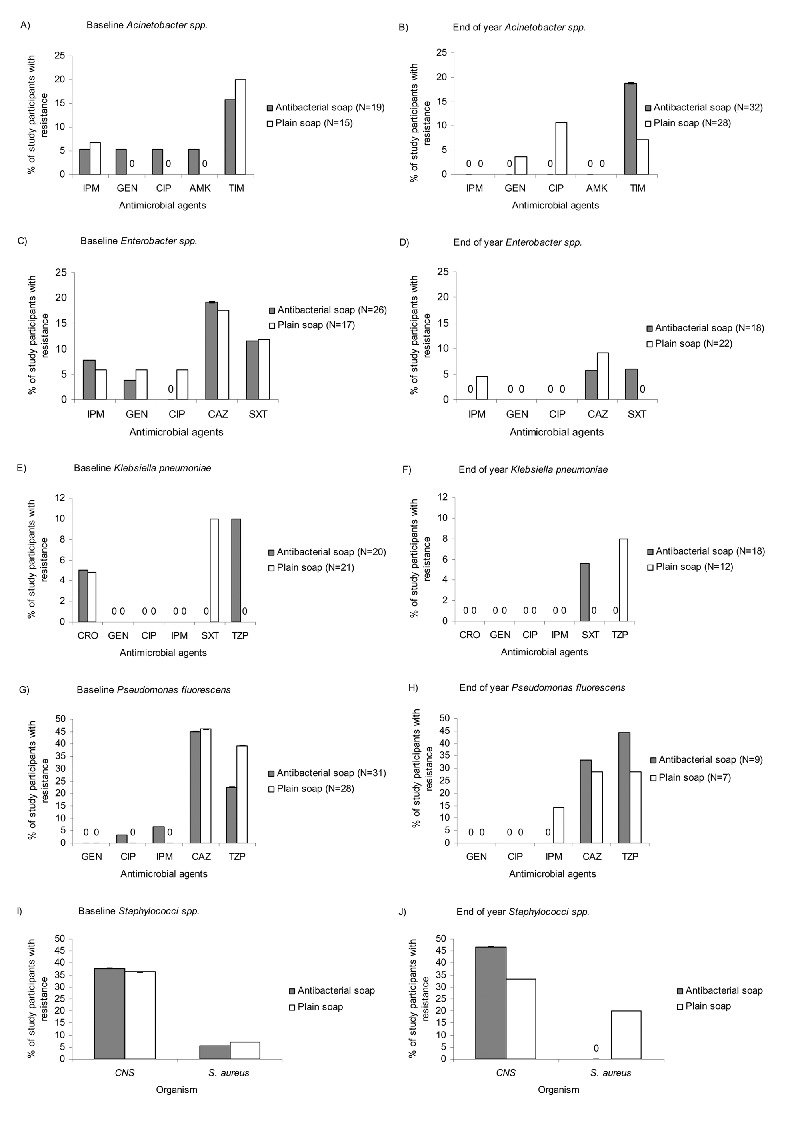
Proportion of study participants with gram-negative bacteria resistant to antimicrobial agents, methicillin-resistant Staphylococcus aureus (MRSA), and methicillin-resistant, coagulase-negative staphylococci (MRCNS). For A and B, Acinetobacter baumanii and A. lwoffi were combined to represent Acinetobacter spp. For C and D, Enterobacter cloacae and E. agglomerans were combined to represent Enterobacter spp. IPM, imipenem; GEN, gentamicin; CIP, ciprofloxacin; AMK, amikacin; CAZ, ceftazidime; TIM, ticarcillin-clavulanic acid; SXT, trimethoprim-sulfamethoxazole; CRO, ceftriaxone; TZP, piperacillin-tazobactam.

GNB and staphylococci were recovered from 164 participants at baseline and 201 participants at year-end. None of the measured demographic and hygiene characteristics differed significantly between the randomized groups (all p>0.10) (Tables 1 and 2). When comparing isolates from the antibacterial users and nonantibacterial users (Figure 2 and Figure A1), no significant differences in the proportions of resistance were found in all species combined or within single species (all p>0.05).

The odds of carrying >1 antimicrobial drug–resistant strain(s) among antibacterial product users and nonusers were not significant at baseline (OR 0.97, 95% CI 0.50–1.89) or after 1 year of antibacterial product use (OR 1.33, 95% CI 0.74–2.41) (Table 3). In addition, the odds of carrying >1 organism with high triclosan MIC among antibacterial product users or nonusers were similar at baseline (OR 1.59, 95% CI 0.84–3.01) and at year-end (OR 1.73, 95% CI 0.97–3.09).

**Table 3:** Logistic regression models for examining factors associated with carriage of organisms with antimicrobial resistance or increased triclosan MICs*

Outcome 1 (>1 organism with resistance to antimicrobial agents on hand)	OR	95% CI , p value	aOR†	95% CI, p value
Baseline characteristics (N = 164)				
Antibacterial product use in household‡	1.16	0.62–2.17, 0.63	0.97	0.50–1.89, 0.91
Observed no. of seconds for handwash by primary caregiver	1.05	1.01–1.09, 0.01	1.05	1.01–1.09, 0.01
Above average log total CFU on hands of primary caregiver after handwash	2.06	1.08–3.93, 0.03	1.81	0.93–3.52, 0.08
Reported no. of hands washes per day for primary caregiver	1.01	0.97–1.04, 0.74	–	–
>1 household members with job in healthcare or daycare	1.28	0.68–2.40, 0.44	–	–
Year-end characteristics (N = 201)				
Antibacterial product use in household	1.44	0.82–2.52, 0.20	1.33	0.74–2.41, 0.34
Observed no. of seconds for handwash by primary caregiver	1.00	0.97–1.04, 0.91	–	–
Above average log total CFU on hands of primary caregiver after handwash	0.62	0.35–1.98, 0.09	–	–
Reported no. of hands washes per day for primary caregiver	0.94	0.89–0.99, 0.04	0.95	0.89–1.01, 0.10
>1 household members with job in healthcare or daycare	0.51	0.29–0.90, 0.02	0.52	0.29–0.95, 0.04
Outcome 2 (>1 organism with increased triclosan MIC on hand)	OR	95% CI , p value	aOR†	95% CI, p value
Baseline (N = 164)				
Antibacterial product use in household‡	1.59	0.84–3.01, 0.16	–	–
Year-end (N = 201)				
Antibacterial product use in household	1.73	0.97–3.09, 0.06	–	–

*OR, odds ratio; CI, confidence interval; aOR, adjusted odds ratio. †OR adjusted for all variables that were significant in univariate analyses at p<0.05. ‡Prior reported antibacterial product use was controlled for but did not have any effect on the point estimate. Therefore, "group" point estimates reflect use of antibacterial product after randomization.

### Individual and Household Characteristics and Susceptibility

At baseline, primary caregivers with higher than average CFU on their hands were twice as likely to carry antimicrobial drug–resistant organisms (Table 3). A slightly increased risk of carrying antimicrobial drug–resistant organisms occurred among those who washed their hands for a longer duration before the culture sample at baseline (Table 3). However, longer duration of handwashing was not associated with reduced bacterial CFU on hands (OR 1.02, 95% CI 0.99–1.06).

At year-end, both the number of times hands were washed per day and the presence of any household member(s) with a healthcare or daycare occupation were significantly associated with reduced carriage of antimicrobial drug–resistant organisms on hands of the primary caregiver (Table 3). Primary caregivers residing in households with members working in healthcare or daycare were significantly more likely to report above-average number of handwashes per day (OR 3.05, 95% CI 1.71–5.44). None of the other characteristics, such as health conditions or antimicrobial drug use, were significantly associated with carriage at baseline or after 1 year (all p>0.05).

## Discussion

This study is the first randomized intervention study to investigate the relationship between antibacterial cleaning and hygiene product use and antimicrobial drug susceptibility of hand microflora within the community setting. Our earlier research, conducted among the same study population described here, showed that use of antibacterial hand soap containing 0.2% triclosan was no more beneficial than plain soap in reducing infectious illness symptoms or bacterial counts on hands of household members (*4**,**5**,**15*). Several avenues of research have contributed to the view that use of products containing triclosan may foster the emergence of antimicrobial drug– or biocide-resistant organisms. This concern stems from reports that exposure to triclosan can lead to bacterial target mutations conferring cross-resistance to isoniazid and selects for mutants bearing resistance to various antimicrobial agents through expression of multidrug-resistant efflux pumps (*12**,**16*). Our findings suggest that household use of antibacterial cleaning and hygiene products for a 1-year period is not a significant risk factor for increasing antimicrobial drug–resistant organisms on the hands of persons in the home.

Few data compare resistance patterns among hand microflora and susceptibility to antibacterial handwashing ingredients. One recent cross-sectional study (*17*) reported a higher prevalence of decreased susceptibility to triclosan among methicillin-resistant *S. epidermidis* compared to methicillin-sensitive *S. epidermidis* clinical isolates. The findings reported in other cross-sectional studies have mainly examined environmental and clinical isolates of bacteria, and the correlations reported have been inconsistent (*11**,**13**,**18**–**20*).

### Other Factors Associated with Antimicrobial Drug Resistance

Several hygiene-related factors were significantly associated with carriage, regardless of antibacterial product use. Longer handwashes were slightly associated with increased risk for carriage of antimicrobial drug–resistant species at baseline; as reported previously, these findings may be an artifact of sampling technique (*5*). The culture was taken directly after the handwash; an increased duration of the wash may have allowed greater dispersal of bacteria into the culture bag.

Primary caregivers residing in households with healthcare or daycare workers had significantly fewer antimicrobial drug–resistant organisms on their hands. This association appears to be influenced by above-average number of handwashes per day by the primary caregiver and indicates that hygiene, regardless of antibacterial ingredients, may reduce household transmission of antimicrobial drug–resistant bacteria.

### Limitations for Detecting Changes in Resistance

A factor that might have attenuated the associations found in this study is a higher baseline level of antimicrobial drug resistance in this community. Higher baseline levels would make detecting small changes in susceptibility attributed solely to use of antibacterial cleaning and hygiene products more difficult. Most persons from our study population were from the Dominican Republic, a country that provides over-the-counter access to antimicrobial agents. In an earlier study within this same community, antimicrobial agents were taken by 354 (39%) of 911 persons reporting infectious disease symptoms within the previous 30 days, which suggests high levels of use (*21*). In addition, this study was conducted for a 1-year period and therefore may not adequately reflect the time-course for development of resistance attributable to use of antibacterial products. Changes in antimicrobial drug resistance during the 1-year period might have been lower than the level of detection that this study was statistically powered to identify. This study was designed to detect an OR >2.11 after 1 year of use, given a power of 80% and a 2-sided α level of 0.05.

Although triclosan susceptibility was examined among various species, we were not able to evaluate potential mechanisms for cross-resistance, such as overexpression of efflux pumps. In addition, when we examined the association between use of antibacterial cleaning and hygiene products and antimicrobial drug resistance, the definition of resistance (>1 organism[s] with antimicrobial drug resistance) did not allow exploration of the potential association with each separate species or antimicrobial drug tested. However, the purpose of our study was to examine overall trends and shifts in antimicrobial drug resistance attributed to the use of antibacterial cleaning and hygiene products, given that the effects of these products in the community are relatively unexplored.

## Conclusion

Currently, no evidence suggests that use of antibacterial soap containing 0.2% triclosan provides a benefit over plain soap in reducing bacterial counts and rate of infectious symptoms in generally healthy persons in the household setting (*4**,**5**,**15*). Our 1-year randomized community intervention study adds to these earlier findings by assessing the potential risks associated with antibacterial product use in the home. The results from our study do not implicate use of antibacterial cleaning and hygiene products as an influential factor in carriage of antimicrobial drug–resistant bacteria on the hands of household members. Although we did not observe a significant impact on antimicrobial drug resistance during the 1-year period, a longer duration and more extensive use of triclosan might provide a suitable environment for emergence of antimicrobial drug–resistant species in the community setting. Further surveillance for the effect of long-term use of antibacterial cleaning and hygiene products on antimicrobial drug resistance in the community is needed.
